# Cytoplasmic Male Sterility-Associated Mitochondrial Gene *orf312* Derived from Rice (*Oryza sativa* L.) Cultivar Tadukan

**DOI:** 10.1186/s12284-021-00488-7

**Published:** 2021-05-22

**Authors:** Ayumu Takatsuka, Tomohiko Kazama, Kinya Toriyama

**Affiliations:** 1 Graduate School of Agricultural Science, Tohoku University, 468-1 Aramaki Aza Aoba, Aoba-ku, Sendai, Miyagi 980-8572 Japan; 2 Present address: Faculty of Agriculture, Kyushu University, 744 Motooka, Nishi-ku, Fukuoka, Fukuoka 819-0385 Japan

**Keywords:** Cytoplasmic male sterility, Mitochondrial gene, *Oryza sativa*

## Abstract

**Background:**

Cytoplasmic male sterility (CMS) is a trait associated with non-functional pollen or anthers, caused by the interaction between mitochondrial and nuclear genes.

**Findings:**

A Tadukan-type CMS line (TAA) and a restorer line (TAR) were obtained by successive backcrossing between the *Oryza sativa* cultivars Tadukan (a cytoplasmic donor) and Taichung 65 (a recurrent pollen parent). Using Illumina HiSeq, we determined whole-genome sequences of the mitochondria of TAA and screened the mitochondrial genome for the presence of *open reading frame* (*orf*) genes specific to this genome. One of these *orf* genes, *orf312*, showed differential expression patterns in TAA and TAR anthers at the meiotic and mature stages, with transcript amounts in TAR being less than those in TAA. The *orf312* gene is similar to the previously described *orf288*, a part of which is among the components comprising *WA352*, a chimeric CMS-associated gene of wild-abortive-type CMS.

**Conclusions:**

The *orf312* gene is a promising candidate for CMS-associated gene in TAA.

**Supplementary Information:**

The online version contains supplementary material available at 10.1186/s12284-021-00488-7.

## Background

Hybrid breeding is an effective approach for enhancing crop yield, including that of rice. To obtain F_1_ hybrid seeds, male-sterile plants are often used as female parents to prevent self-pollination, and most commercial hybrid rice varieties have been developed utilizing cytoplasmic male sterility (CMS) and photoperiod/temperature-sensitive genic male sterility techniques (Huang et al. 2014). CMS occurs due to an interaction between mitochondrial and nuclear genomes. In this regard, the expression of certain mitochondrial *open reading frame* (*orf*) genes, which are referred to as CMS-associated genes, is known to cause dysfunctions in pollen development (Huang et al. 2014). The expression of CMS-associated *orf*s is in turn often suppressed by the expression of particular nuclear-encoded genes, referred to as the *RESTORER OF FERTILITY* (*RF*) genes, which rescue pollen development, resulting in the recovery of fertility. Most *RF* genes cloned to date have been reported to encode pentatricopeptide repeat (PPR) proteins, which are RNA-binding proteins involved in RNA processing in mitochondria and plastids (Schmitz-Linneweber and Small 2008).

In rice, the following CMS-associated *orf*s have been thus far reported: *orf79* for Chinsurah-Boro-type CMS (BT-CMS) and Lead rice-type CMS (LD-CMS) (Iwabuchi et al. 1993; Akagi et al; 1994, Itabashi et al. 2009), *orfH79* for Honglian-type CMS (HL-CMS) (Yi et al. 2002), *orf352/WA352* for wild-abortive-type CMS (WA-CMS) (Luo et al. 2013) and RT102-type CMS (Okazaki et al. 2013), *orf307* for Chinese wild rice-type CMS (CW-CMS) (Fujii et al. 2010), *orf113* for RT98-type CMS (Igarashi et al. 2013), and *orf182* for Dongxiang-type CMS (D1-CMS) (Xie et al. 2018). In recent years, the identification of CMS-associated *orf*s has primarily been achieved based on whole-genome sequencing and through searches for specific *orf*s that are not found in other CMS or maintainer cultivars. Typically, a CMS-associated *orf* carries a fragment of coding or flanking sequences of known mitochondrial genes, encodes a protein with transmembrane domains, and often exhibits differential expression patterns in the absence or presence of the corresponding *RF* gene (Iwabuchi et al. 1993; Akagi et al. 1994; Yi et al. 2002; Fujii et al. 2010; Igarashi et al. 2013; Okazaki et al. 2013; Luo et al. 2013; Xie et al. 2018).

A typical example of CMS-associated *orf*s in rice is *orf79*, which has been reported to have a chimeric structure incorporating a sequence similar to that of *cox1*, a gene that encodes cytochrome *c* oxidase subunit 1, and that of a gene of unknown origin, encoding a peptide with a transmembrane domain. *orf79* is co-transcribed with the *atp6* gene, which encodes ATP synthase F_0_ subunit 6. The transcripts of *atp6-orf79* are cleaved by the action of the *Rf1a* gene, which encodes a pentatricopeptide repeat (PPR)-containing protein (Kazama et al. 2008). The *orf352/WA352* sequence (Okazaki et al. 2013; Luo et al. 2013) includes a partial 5′-terminal sequence of the Nipponbare *orf284* sequence (100% nucleotide identity over 286 bp), a sequence of unknown origin, a sequence homologous to the central part of the Nipponbare *orf224* sequence (78% nucleotide identity over 68 bp), and a partial 3′-terminal sequence of the *orf288* sequence (97% identity over 557 bp), where Nipponbare *orf284*, *orf224*, and *orf288* encode hypothetical proteins (GenBank PROTEIN_ID AAZ99359.1, AAZ99350.1, AAZ99379.1, respectively). *orf352/WA352* is co-transcribed with the upstream gene *rpl5*, which encodes ribosomal protein large subunit 5. The transcripts of *rpl5* and *orf352/WA352* disappear in the presence of the *Rf4* gene, which encodes a PPR protein (Luo et al. 2013; Kazama and Toriyama 2014).

With an aim to identify new CMS lines in rice, we produced several cytoplasm-substitution lines of the *japonica* group cultivar Taichung 65 (T65) by successive backcrossing, where different rice cultivars were used as the initial female parent and T65 as a recurrent pollen parent. During this process, we identified a CMS line in the backcross progeny derived from an *indica* group cultivar, Tadukan, from the Philippines. A CMS line with Tadukan cytoplasm was reported as early as 1962 (Kitamura 1962a; b), in which sterility was found to be associated with a defect in anther dehiscence, although no studies on the morphology of the pollen of this line have been reported. Subsequently, this CMS line was named [*cms-TA*] (Kinoshita 1997). With respect to CMS, the gene name and the gene symbol assigned by the Committee on Gene Symbolization, Nomenclature, and Linkage (CGSNL) are *CYTOPLASMIC MALE STERILITY TA* and [*CMS-TA*] (Oryzabase; https://shigen.nig.ac.jp/rice/oryzabase/locale/change?lang=en), respectively. The feature of Tadukan-type CMS with defective pollen dispersal is quite unique, as other CMS types so far reported in rice show pollen-defective phenotypes (Huang et al. 2014). To date, however, there have been no related molecular analyses.

In the present study, to provide new insights for the molecular mechanism of this unique Tadukan-type CMS exhibiting anther indehiscence, we sought to identify CMS-associated candidate genes in [*CMS-TA*] mitochondria using a next-generation sequencing approach and accordingly detected a specific *orf*, which we named *orf312.* Northern blot analysis of anther RNA revealed that the expression levels of this gene are affected by the presence of the *Rf* gene derived from Tadukan, thereby indicating that *orf312* could be a strong candidate for Tadukan-type CMS.

## Results

### Pollen and Anther Phenotypes

**Fig. 1:**
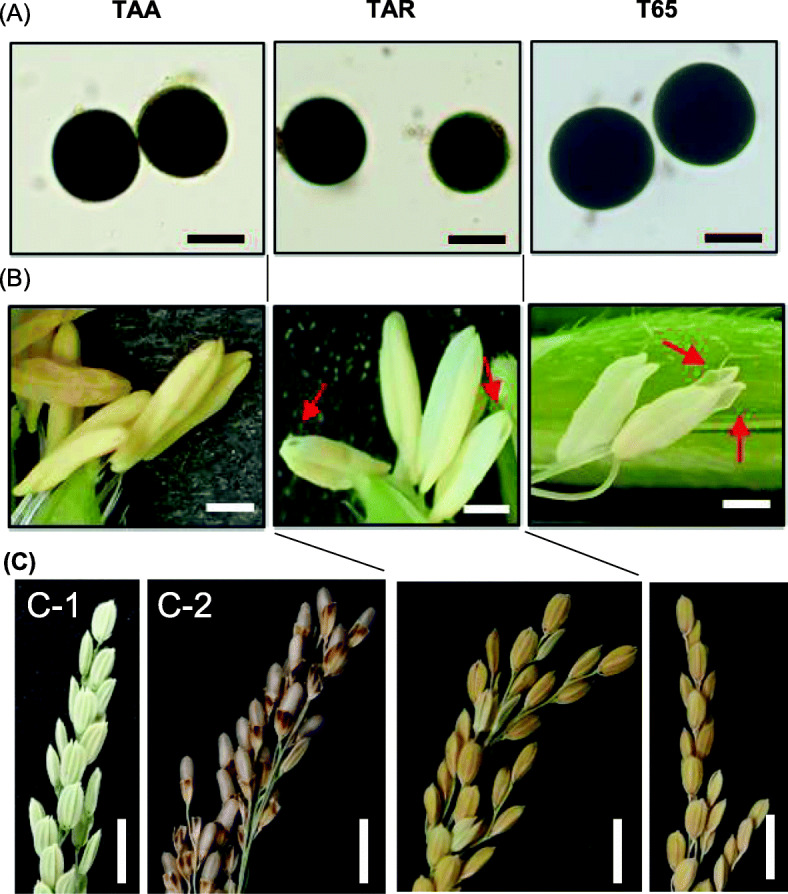
The pollen, anthers and seed setting of TAA, TAR, and Taichung 65 (T65). **a** Mature pollen stained with I_2_-KI (Bar = 25 μm). **b** Anthers at the flowering stage. (Bar = 1 mm). Arrows indicate dehiscence of anthers. **c** Seed setting of bagged panicles. Panel C-1 indicates no seed setting without artificial pollination, while panel C-2 indicates seeds on clipped spikelets after compulsive self-pollination of TAA (Bar = 1 cm)

Observations through an optical microscope revealed that pollen grains of the Tadukan-type CMS line TAA were morphologically normal and stained darkly with I_2_-KI, similar to those of the maintainer line T65 and the fertility restorer line TAR (Fig. 1a). In TAA, however, we found that the anthers failed to dehisce, and no pollen was scattered after flowering, whereas pollen grains were released from widened slits in the apical and basal parts of the anthers of TAR and T65 (Fig. 1b). The average seed setting rates of the bagged panicles for TAA, TAR, and T65 were 0%, 75.0%, and 85.2%, respectively (Table S1 and Fig. 1c). To examine the pollen viability of TAA, the pollen grains were squeezed out of the anthers with forceps and deposited on the stigmas. This compulsive self-pollination resulted in seed setting in the same manner as we usually obtained by the artificial hand pollination (Fig. 1c), indicating that the pollen grains themselves were viable.

### Sequence Assembly and the Search for Specific *orf*s

**Fig. 2:**
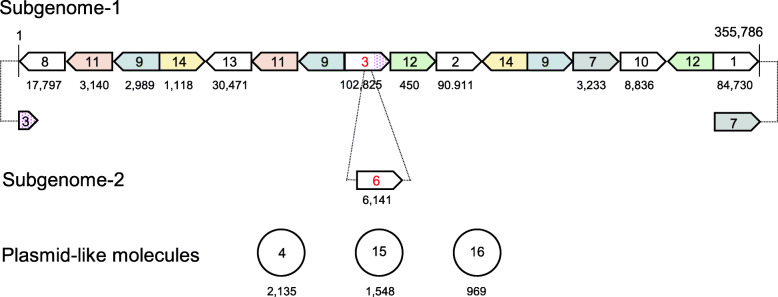
Configuration of contigs in the TAA mitochondrial genome. The same contigs are indicated by the same colors. Numbers within the boxes and circles indicate the names of contigs, and the numbers below each box and circle indicate the contig lengths. Box and circle sizes are not to scale. Connections of the end of subgenome-1 and subgenome-2 are indicated by dotted lines

The TAA mitochondrial genome was assembled into 16 contiguous sequences (contigs_1 to _16; Fig. 2 and Table S2). A BLAST search against DNA databases revealed that contig_4 (2135 bp) was 99% identical to the mitochondrial plasmid-like molecule B1 (DDBJ accession D00293), contig_15 (1548 bp) was 99% identical to B2 (DDBJ accession X16153), and contig_16 (969 bp) was 99% identical to B4 (DDBJ accession X07904). B1, B2, B3 and B4 are plasmid-like small circular molecules that have been previously reported in Boro-Taichung-type CMS (Kazama and Toriyama 2016). In the present study, however, we were unable to detect the B3 molecule in the TAA mitochondrial genome. The other 13 contigs were manually assembled based on the overlapped 98-bp sequences at the end of each contig, such that each contig appeared at least once when constructing the smallest potential genome. The linkage of each contig was validated using PCR analysis (Fig. S1). Except for contig_6, the remaining 13 contigs were assembled into a 355,786-bp linear molecule and named subgenome-1 (Fig. 2). One end, which consisted of contig_1, could be connected to contig_7 (Fig. S1). In contrast, the other end containing contig_8 did not show connections to any other contigs. Sequences adjacent to the end of conting_8 were amplified with eight arbitrary primers (FP1 to FP8) using FPNI-PCR and we accordingly obtained bands, ranging in size from 0.4 to 3 kbp, on the 3rd nested PCR (Fig. S2). No corresponding bands were detected when T65 was used as a template. DNA was extracted from each of seven bands, and the nucleotide sequences (approximately 0.3 kb long from the end) were determined. The sequences of all seven bands, determined from the direction of Contig_8-specific primers (SP3), were found to be identical to sequences corresponding to the region from positions 153,932 to 154,248 in subgenome-1 (Fig. S2). These results indicate that contig_8 is connected to the middle of contig_3 (Fig. 2). Contig_5 (212 bp) was included within contig_2 and contig_13 (Table S2). These results thus indicate that alternative assembly configurations are also possible. For contig_6 (6141 bp), we were unable to identify any adjacent contigs and thus designated this contig as subgenome-2 (Fig. 2). All known mitochondrial genes in Nipponbare, except for *orf288* and *orf194* (encoding hypothetical proteins; GenBank Protein_ID AAZ99379.1 and AAZ99346.1, respectively) were mapped on subgenome-1 (Fig. S3).

Subgenome-1 and subgenome-2 (= contig_6) were subsequently screened for the presence of specific *orf*s that encoded proteins of more than 70 amino acids, and that which were not present in the mitochondrial genome of the *japonica* group standard rice cultivar Nipponbare or an *indica* group maintainer line for WA-CMS that did not exhibit CMS. We identified two such *orf*s in subgenome-2, which were named *orf312* and *orf115* based on the number of amino acids that each was predicted to encode (Table S3). We then investigated whether these *orf* genes were present in the mitochondrial genomes of other CMS-type lines (BT, LD, CW, RT98, and RT102). We thus identified an *orf* completely identical to *orf115* in the mitochondrial genomes of the BT-, LD-, and RT98-type CMS lines, whereas *orf312* was found to be unique to TAA. The *orf312* gene was accordingly selected as a candidate for a TAA-type CMS-associated gene.

**Fig. 3:**
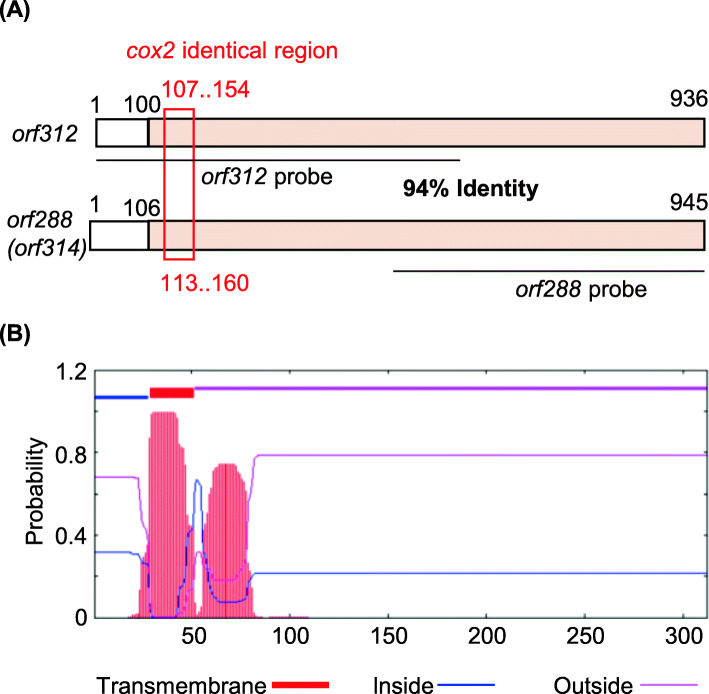
Schematic structure of the CMS-associated gene *orf312*. **a** Similarities of the *orf312* gene of TAA and the *orf288* gene of Nipponbare. The pink region (100th to 936th nucleotides) shows 94% identity. **b** The location and probability associated with the predicted transmembrane domain of ORF312

*orf312* was found to be relatively similar to the *orf288* gene in Nipponbare, showing an overall identity of 90%, and 94% identity in the sequence encompassing the 100th to 936th nucleotides (Fig. 3a). Although *orf288* was originally characterized as encoding a hypothetical protein (GenBank Protein ID: AAZ99379), it has more recently been reported to encode 314 amino acids and renamed *orf314* (Tang et al. 2017). However, for the current purposes, we have continued to refer to the gene as *orf288*, given that sequences of the gene in mitochondrial genomes maintained in DNA databases still retain this name. The *orf288* gene contains a 48-bp fragment identical to the sequence of exon 1 of *cox2*, which encodes subunit 2 of cytochrome *c* oxidase (Fig. 3a); however, its function has yet to be ascertained. The amino acid identity between *orf312* and *orf288* is 91%, and in the ORF312 protein, we predicted one transmembrane domain in the N-terminal region (29th to 55th amino acid position) corresponding to the *cox2*-identical region (Fig. 3b).

**Fig. 4:**
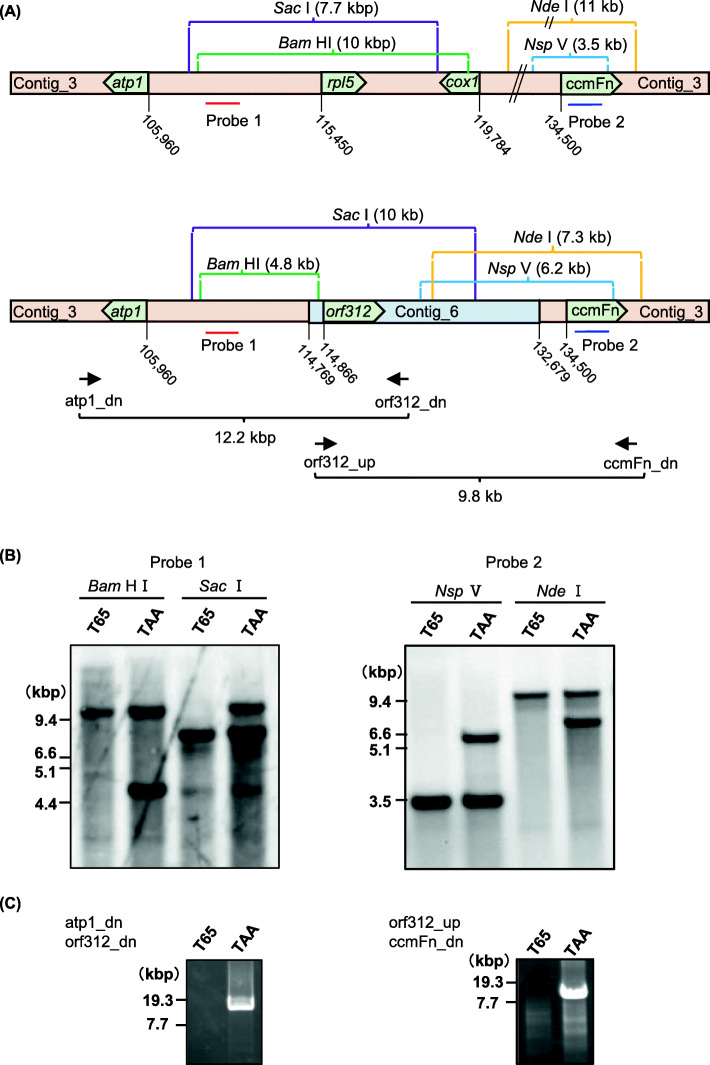
Schematic genomic structure around *orf312*. **a** Structure of contig_3 and linkage between contig_6 and contig_3. Nucleotide positions in subgenome-1 are indicated. **b** Southern blot analysis performed to detect the presence of the two genomic structures shown in panel A. The probes are indicated in panel A. **c** PCR performed to detect the linkages between *orf312* and *atp1* or *ccmFn*

The *orf312* gene is located near the end of contig_6 (6141 bp in length) and the 5′-upstream region of the gene is restricted to 102 bp. Sequences adjacent to the end of conting_6 were amplified with eight arbitrary primers (FP1 to FP8) using FPNI-PCR, and we accordingly obtained a single band, ranging in size from 0.4 to 1.2 kbp, when using primers FP3, FP4, FP6, FP7 and FP8, and two bands with FP5 on the 3rd nested PCR (Fig. S4). No corresponding bands were detected when T65 was used as a template. DNA was extracted from each of these bands, and the nucleotide sequences (approximately 200 to 400 bp long from the end) were determined. The sequences of all seven bands, determined from the direction of gene-specific primers (SP2 and SP3), were found to be identical to sequences in contig_3, corresponding to the region from positions 114,538 to 114,770 in subgenome-1 (Fig. S4). Sequences at the opposite end, obtained using SFP2 primers, were also found to be identical sequences in contig_3 from positions 113,782 to 113,998 in subgenome-1 (Fig. S4). Moreover, we established that the nucleotide sequence from positions 1 to 98 in contig_6 is completely identical to that of contig_3, which corresponds to 114,769 to 114,866 in subgenome-1 (Fig. 4a). Sequences adjacent to the 3′ end of conting_6 were also determined by FPNI-PCR. The nucleotide sequences of six bands obtained using arbitrary primers FP2, FP3, FP4, FP5, FP7 and FP8 were identical to sequences in contig_3 from positions 132,623 to 132,882 in subgenome-1 (Fig. S4). These results accordingly indicate that contig_6 is located within the central region of contig_3, substituting contig_6 (6141 bp in length) for a 17,911-bp fragment of contig_3 (position 114,769 to 132,679 in subgenome-1), as depicted in Fig. 4a.

The presence of a molecule corresponding to the identified sequence was confirmed by Southern blot analysis. Connection of 5′-side of conting_6 was confirmed using the probe 1 designed upstream of contig_6. We detected 4.8-kbp *Bam*HI and 10-kbp *Sac*I fragments, as predicted from the results of FPNI-PCR, together with 10-kbp *Bam*HI and 7.7-kbp *Sac*I fragments predicted from contig_3 (Fig. 4b). Connection of 3′-side of conting_6 was confirmed using the probe 2 designed downstream of contig_6. We detected 6.2-kbp *Nsp*V and 7.3-kbp *Nde*I fragments, as predicted from the results of FPNI-PCR, together with 3.5-kbp *Nsp*V and 11-kbp *Nde*I fragments predicted from contig_3 (Fig. 4b). These connections were also confirmed using long-PCR primers designed from sequences downstream of *atp1* encoding ATP synthase subunit 1 and *ccmFn* encoding cytochrome c biogenesis Fn (Fig. 4c). These results thereby revealed the presence of two types of molecules in TAA (Fig. 4a). The almost identical signal intensities of the two bands indicated that these molecules are present in equivalent amounts in the mitochondria of TAA. In contrast, we detected only a single band in T65, indicating the presence of a molecular type corresponding to contig_3 (Fig. 4b).

**Fig. 5:**
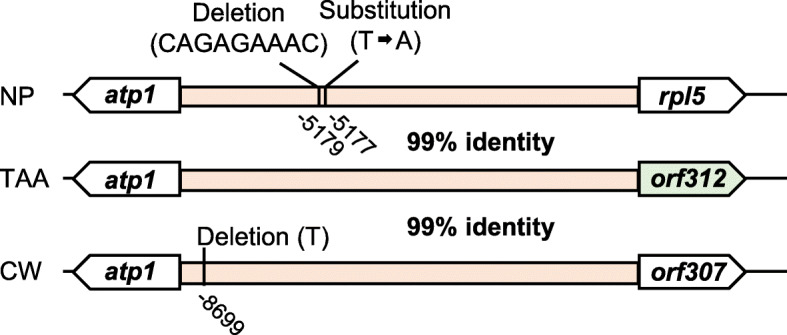
Schematic structures of the upstream region of *orf312* and homologous regions in Nipponbare (NP) and CW-type CMS (CW). Nucleotide identities are shown for the pink region. Substituted and deleted nucleotides are indicated with nucleotide positions from the start codon of *rpl5* or *orf307*

The *atp1* gene, encoding ATP synthase subunit 1, is shown to lie 8.9 kbp upstream of the *orf312* gene (Fig. 4). The intergenic region between *atp1* and *orf312* was found to be comparable to that between *atp1* and *rpl5*, the latter of which encodes ribosomal protein large subunit 5 in Nipponbare, with 99% identity, thereby indicating that the *orf312* gene has a promoter region identical to that of *rpl5* (Fig. 5). This region is also similar to the intergenic region between *atp1* and the CMS-associated gene *orf307* in CW-type CMS, showing 99% identity (Fig. 5).

### Northern Blot Analysis of *orf312*

**Fig. 6:**
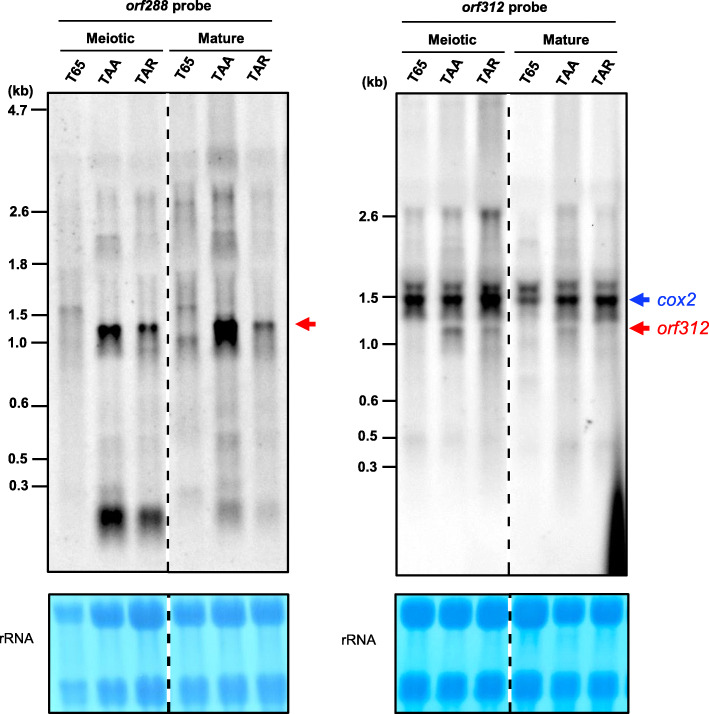
Northern blot analysis of RNA isolated from spikelets of TAA, TAR and Taichung 65 (T65) at the meiotic stage and anthers at the mature stage. The probes are shown in Fig. 3a. Dashed lines indicate where intervening lanes were removed for clarity. Methylene blue staining of rRNA is shown as a loading control

In general, CMS-associated genes are expected to show contrasting expression patterns in the presence or absence of an *Rf* gene. To assess whether *orf312* exhibits such differential expression, we performed northern blot analysis on RNA obtained from spikelets at the meiotic stage and anthers at the mature stage. A probe was prepared from half of the *orf288* sequence, in which the nucleotide sequence was 88% identical to that of *orf312*, excluding a region identical to *cox2*. We accordingly detected a 1.2-kb band for TAA and TAR, whereas no comparable signal was obtained for T65 (Fig. 6 and Fig. S5). This band was considered to correspond to the *orf312* transcripts cross-reacted with the *orf288* probe, because *orf288* was not present in the mitochondrial genome of Tadukan and *orf312* was the unique sequence sharing homology with the *orf288* probe. Moreover, we found that the signal intensity of the TAR band for mature-stage anthers was weaker than that of TAA. Northern blot analysis was also carried out using the *orf312* probes including a region identical to *cox2,* which detected the 1.5-kb *cox2* transcripts as well as the 1.2-kb *orf312* transcripts (Fig. 6 and Fig. S5)*.* Although the signal intensities of the *cox2* transcripts were identical between TAA and TAR, those of *orf312* transcripts in TAR were always greatly reduced compared to those in TAA at the meiotic and mature anther stages on six independent northern blots (Fig. 6 and Fig. S5). These results thus tend to indicate that *orf312* RNA accumulation is reduced by the action of an *Rf* gene derived from Tadukan.

## Discussion

### Pollen and Anther Phenotypes

To date, more than 30 types of CMS have been recorded based on cytoplasmic origin and the relationship between restoration and maintenance (Rice Database Oryzabase; https://shigen.nig.ac.jp/rice/oryzabase/locale/change?lang=en). These are categorized into three main types based on the morphology of abortive pollen (Huang et al. 2014). Wild-abortive-type pollen grains, exemplified by WA-type CMS, are irregular in shape and unstainable in I_2_-KI solution, whereas the BT type, exemplified by BT-CMS and HL-CMS, are spherical and slightly stained with I_2_-KI, and the CW type, exemplified by CW-CMS and RT98-CMS, are morphologically normal but fail to germinate. In the current study of Tadukan-type CMS, the pollen grains were viable, as seeds were set upon compulsive self-pollination, and there appeared a defect in anther dehiscence (Fig. 1). A CMS phenotype characterized by indehiscent anthers is consistent with the findings of a previous study in which Tadukan was backcrossed with the cultivar Norin 8 (Kitamura 1962a). We thus speculate that the failure of anthers to dehisce might be the primary factor contributing to the male sterility of TAA. Cytological studies of anthers should be examined in the future.

### *orf312*: a CMS-Associated Gene

Based on our observations, *orf312* would appear to be a strong candidate for a CMS-associated gene. The amino acid sequence of ORF312 contains a region similar to that of ORF288 (renamed ORF314 by Tang et al. 2017), a part of which is also included in WA352, a product of the CMS-associated gene of WA-CMS (Fig. S6). The WA352 protein has been reported to accumulate in tapetal cells at the microspore mother cell stage and physically interacts with OsCOX11 (subunit 11 of cytochrome *c* oxidase) encoded by a nuclear gene. This interaction inhibits the normal functioning of OsCOX11, thus causing a reactive oxygen species burst and premature programmed cell death (PCD) of the tapetum, and consequently pollen abortion. Two regions of WA352 have been shown to interact with OsCOX11, which we found to be almost identical to the corresponding two regions retained in the ORF312 protein of TAA (Fig. S6). It is therefore conceivable that these regions in ORF312 interact with OsCOX11. However, the mechanisms underlying sterility are assumed to differ. Whereas the pollen grains of WA-CMS are aborted immediately after pollen meiosis, giving rise to the production of shrunken empty pollen grains, those of TAA are engorged with starch and appear morphologically normal until the mature stage.

Our results indicate that ORF312 affects anther dehiscence, which requires the breakdown of cells in the stomium of the anther wall, which undergo PCD (Beers 1997). The release of mature pollen in rice is triggered by the swelling of pollen grains (Matsui et al. 1999), and we speculate that ORF312 might accumulate in the stomium and affect the breakdown of the stomium, or accumulate in pollen grains and thereby suppress pollen grain swelling. It is also conceivable that this protein might accumulate in the anther tapetum and indirectly affect anther dehiscence, as defective tapetum PCD has previously been reported to cause anther indehiscence in an autophagy-deficient mutant of rice (Kurusu et al. 2014). In the present study, however, we did not investigate the accumulation of ORF312 in anthers, although in this regard, work is currently in progress to obtain specific antibodies for detection of the ORF312 protein.

Although *orf288* is present in the T65 mitochondrial genome, as in Nipponbare, its transcripts were scarcely detected by the *orf288* probe in anthers at either the meiotic or mature stage (Fig. 6 and Fig. S5). These observations are consistent with those made in a previous study, in which the authors failed to detect *orf288* transcripts in the anthers and other tissues of Nipponbare (Tang et al. 2017). In contrast, we established that the *orf312* gene is expressed in anthers (Fig. 6), and suspect that the acquisition of anther expression can be attributed to the activity of the promoter region of *rpl5*, which encodes a ribosomal protein (Fig. 5). In this regard, it has previously been demonstrated that the *rpl5* promoter is associated with the transcription of *WA352*, as these two genes are co-transcribed (Luo et al. 2013). Similarly, it has been found that transcription of the CMS-associated gene *orf307* in CW-type CMS is also mediated by the *rpl5* promoter (Fujii et al. 2010).

Recently, we employed mitochondria-targeting TALEN (mitoTALEN) to knock out the CMS-associated gene *orf79* in BT-type CMS rice (Kazama et al. 2019). The mitoTALEN vector is designed to deliver the TALEN protein into mitochondria by the insertion of a mitochondrial-targeting signal sequence, and we found that knocking out the *orf79* gene restored the fertility of flowering plants, thereby demonstrating that mitoTALEN can serve as an effective approach for revealing the role of CMS-associated genes. We are currently using this technique to knock out the *orf312* gene in TAA to establish whether the *orf312* gene is a CMS-causing gene.

## Conclusions

We found a new CMS-associated gene, named *orf312*, in Tadukan-type CMS in rice, which showed anther indehiscence. The *orf312* gene was similar to *orf288* of Nipponbare. Its expression level in anthers was higher than the latter, probably because of the acquisition of the promoter region of *rpl5*. The *orf312* transcripts were decreased in the restorer line. Our results indicate that *orf312* is an excellent candidate gene causing Tadukan-type CMS.

## Materials and Methods

The *Oryza sativa* L. Tadukan cultivar was provided by the National Institute of Agrobiological Sciences Genebank (Tsukuba, Japan) as WRC20 (Kojima et al. 2005). An F_1_ hybrid derived from a cross between the cultivars Tadukan (female) and T65 (male) was successively backcrossed with T65, and the resulting BC_11_F_1_ generation was used as the Tadukan-type CMS line, TAA. A fertility restorer line, TAR, was selected from the BC_3_F_3_ generation, in which SSR marker loci (SSRH10023 and SSRH10045; Igarashi et al. 2016) flanking the *rf1* locus (Os10g0497432) on chromosome 10 carried Tadukan homozygous genotypes, as most of the PPR-type *Rf* genes have been shown to be located in the vicinity of this region (Kazama and Toriyama 2003; 2014; Wang et al. 2006; Tang et al. 2014; Igarashi et al. 2016).

The mature anthers of TAA and TAR were harvested a day prior to anthesis, and the pollen grains therein were stained with an iodine-potassium iodide solution for observations of starch accumulation. Seed setting rates were determined by counting the filled grains and total grains of three bagged panicles. The compulsive self-pollination of TAA was carried out as follows. The anthers were taken from the flowering spikelets and the pollen grains were squeezed out of the anthers with forceps and deposited on the stigmas of other spikelets, which were clipped off by scissors and emasculated a day before pollination.

### Sequencing of the Tadukan Mitochondrial Genome

Mitochondria were isolated from calli obtained from mature seeds of TAA using sucrose gradient centrifugation, as described previously. (Kazama et al. 2008). Mitochondrial DNA was extracted using a DNeasy Plant Mini Kit (Qiagen, http://www.qiagen.com/). Nucleotide sequences of the TAA mitochondrial genome were determined by TaKaRa Bio Inc. (www.takara-bio.com/) with an Illumina HiSeq sequencer using a 100-bp paired-end library and the de novo assembly program Velvet (Zerbino and Birney 2008). A graphical representation of the genome map was generated using OGDraw software (https://chlorobox.mpimp-golm.mpg.de/OGDraw.html; Lohse et al. 2007). Contig linkages were confirmed through PCR using selected primers (Table S4) under the following amplification conditions: 94 °C for 1 min, followed by 30 cycles of 94 °C for 30 s, 57 °C for 30 s, and 72 °C for 30 s, and a final extension at 72 °C for 1 min.

### Search for Specific *orf*s

The *orf*s were identified using Artemis software (Rutherford et al. 2000), in which the threshold for identifying *orf*s was set to 70 amino acids, and common *orf*s showing more than 99% identity to those in Nipponbare (GenBank accession DQ167400) and a maintainer line for WA-CMS (GenBank accession number JF281153) were discarded, as described previously (Fujii et al. 2010, Igarashi et al. 2013, Okazaki et al. 2013, Kazama and Toriyama 2016). The nucleotide sequences of the remaining *orf*s were compared with those of the following CMS lines: BTA (DDBJ accession AP017885 and AP017386), CWA (AP011076), LDA (AP011076), RT98A (AP012527), and RT102A (AP012528).

The TMHMM server v.2.0 (http://www.cbs.dtu.dk/services/TMHMM/; Krogh et al. 2001) was used to predict transmembrane helices in proteins encoded by the detected *orf*s.

### Determination of Adjacent Sequences

Genomic DNA was isolated from the leaf blades of Tadukan and T65 plants using a DNeasy Plant Mini Kit (Qiagen). Adjacent unknown sequences linked to the end of contig_8 and conting_6 were determined using a method of fusion primer and nested integrated PCR (FPNI-PCR), which is a modified version of thermal asymmetric interlaced (TAIL)-PCR, as described by Wang et al. (2011) and Kazama et al. (2019). The primers used for amplification are listed in Table S4. The nucleotide sequences were determined by direct sequencing of the PCR products using a CEQ8000 genetic analysis system (Beckman Coulter, https://www.beckmancoulter.com/).

Southern blot analysis was carried out using the method described by Kazama and Toriyama (2016). DNA was extracted using a DNeasy Plant Mini Kit (Qiagen). To investigate the linkage between subgenome-1 and contig_6, we digested 1 μg of total DNA with *Bam*HI, *Sac*I, *Nde*I or *Nsp*V. Probes were produced using PCR DIG Labeling Mix (Roche, https://www.roche.com), with the primers listed in Table S4. PCR detection of the linkage between *orf312* and *atp1* or *ccmFn* was carried out using primers listed in Table S4 and Tks Gflex DNA Polymerase (TaKaRa Bio) under the following conditions: 94 °C for 1 min, followed by 30 cycles of 98 °C for 10 s, 60 °C for 15 s, and 68 °C for 6 min.

### Northern Blot Analysis

Total RNA was isolated from spikelets containing anthers at the meiotic stage or from mature anthers using a RNeasy Plant Mini Kit (Qiagen). Three micrograms of each type of RNA were subjected to northern blot analysis as previously described (Kazama et al. 2008). The *orf288* probe and the *orf312* probe were synthesized using PCR DIG labeling Mix (Roche) with the genomic DNA of T65 and Tadukan, respectively, and the primers listed in Table S4. Signals were detected using a LAS-4000 mini image analyzer.

## Supplementary Information

**Additional file 1: Table S1.** The seed setting rate of TAA, TAR, and Taichung 65 (T65). **Table S2.** The position of contigs in TAA mitochondrial subgenome-1 and -2, and plasmid-like molecules. **Table S3.** TAA-specific *open reading frames* (*orf*s). **Table S4.** The sequences of primers (5′–3′) used in this study

**Additional file 2: Fig. S1.** Validation of the contig linkage of subgenome-1 based on PCR analysis. Lane numbers refer to the linkages between different contigs. **Fig. S2.** Determination of the adjacent sequences of contig_8. **(A)** Amplification of the region adjacent to contig_8 using FPNI-PCR. **(B)** Schematic structures of the PCR products indicated by stars in panel A. The blue color indicates the regions in which nucleotide sequences were determined. The nucleotide sequences coincide with those in subgenome-1. **Fig. S3.** A hypothetical master molecule of subgenome-1 of the TAA mitochondrial genome. The graphical representation of the genome map was generated using OGDraw software. **Fig. S4.** Determination of the upstream and downstream regions of contig_6. **(A)** Amplification of the region adjacent to contig_6 using FPNI-PCR. **(B)** Schematic structures of the PCR products indicated by stars in panel A. The blue color indicates the regions in which nucleotide sequences were determined. The nucleotide sequences coincide with those in subgenome-1. **Fig. S5.** Reproducibility of northern blot analysis of RNA isolated from spikelets of TAA, TAR and Taichung 65 (T65) at the meiotic stage and anthers at the mature stage. The probes are shown in Fig. 3a. Dashed lines indicate where intervening lanes were removed for clarity. Methylene blue staining of rRNA is shown as a loading control. **Fig. S6.** Comparison of the amino acid sequences of ORF288 (encoding 314 amino acids) in Nipponbare, ORF312 in TAA and WA352 in WA-CMS using ClustalW (https://clustalw.ddbj.nig.ac.jp). The COX11-interaction regions reported for WA352 are highlighted. Amino acids in ORF312 that differ from those in WA352 in these regions are indicated by red color.

## Data Availability

The datasets supporting the conclusions of this article are included within the article. The nucleotide sequences of the subgenome-1, subgenome-2, B1-like, B2-like and B4-like molecules of TAA mitochondrial genome have been deposited to DNA Data Bank of Japan under the accession numbers of LC595639 and LC592696 to LC592699.

## Abbreviations

BT-CMS: Chinsurah Boro-type CMS
CMS: Cytoplasmic male sterility
CW-CMS: Chinese wild rice-type CMS
*Rf*: *Restorer of fertility*
PPR: Pentatricopeptide repeat
T65: Taichung 65
TAA: Tadukan-type CMS line
TAR: Tadukan-type restorer line
WA-CMS: Wild-abortive-type CMS
