# Supplementary material for: Cytoplasmic Male Sterility-Associated Mitochondrial Gene orf312 Derived from Rice (Oryza sativa L.) Cultivar Tadukan
Source: Rice (N Y). 2021 May 22;14:46. doi: 10.1186/s12284-021-00488-7 (PMC8141088; doi:10.1186/s12284-021-00488-7)
Supplement: Supplementary file 2 — Additional file 2: Fig. S1. Validation of the contig linkage of subgenome-1 based on PCR analysis. Lane numbers refer to the linkages between different contigs. Fig. S2. Determination of the adjacent sequences of contig_8. (A) Amplification of the region adjacent to contig_8 using FPNI-PCR. (B) Schematic structures of the PCR products indicated by stars in panel A. The blue color indicates the regions in which nucleotide sequences were determined. The nucleotide sequences coincide with those in subgenome-1. Fig. S3. A hypothetical master molecule of subgenome-1 of the TAA mitochondrial genome. The graphical representation of the genome map was generated using OGDraw software. Fig. S4. Determination of the upstream and downstream regions of contig_6. (A) Amplification of the region adjacent to contig_6 using FPNI-PCR. (B) Schematic structures of the PCR products indicated by stars in panel A. The blue color indicates the regions in which nucleotide sequences were determined. The nucleotide sequences coincide with those in subgenome-1. Fig. S5. Reproducibility of northern blot analysis of RNA isolated from spikelets of TAA, TAR and Taichung 65 (T65) at the meiotic stage and anthers at the mature stage. The probes are shown in Fig. 3a. Dashed lines indicate where intervening lanes were removed for clarity. Methylene blue staining of rRNA is shown as a loading control. Fig. S6. Comparison of the amino acid sequences of ORF288 (encoding 314 amino acids) in Nipponbare, ORF312 in TAA and WA352 in WA-CMS using ClustalW (https://clustalw.ddbj.nig.ac.jp). The COX11-interaction regions reported for WA352 are highlighted. Amino acids in ORF312 that differ from those in WA352 in these regions are indicated by red color. [file 12284_2021_488_MOESM2_ESM.pdf]

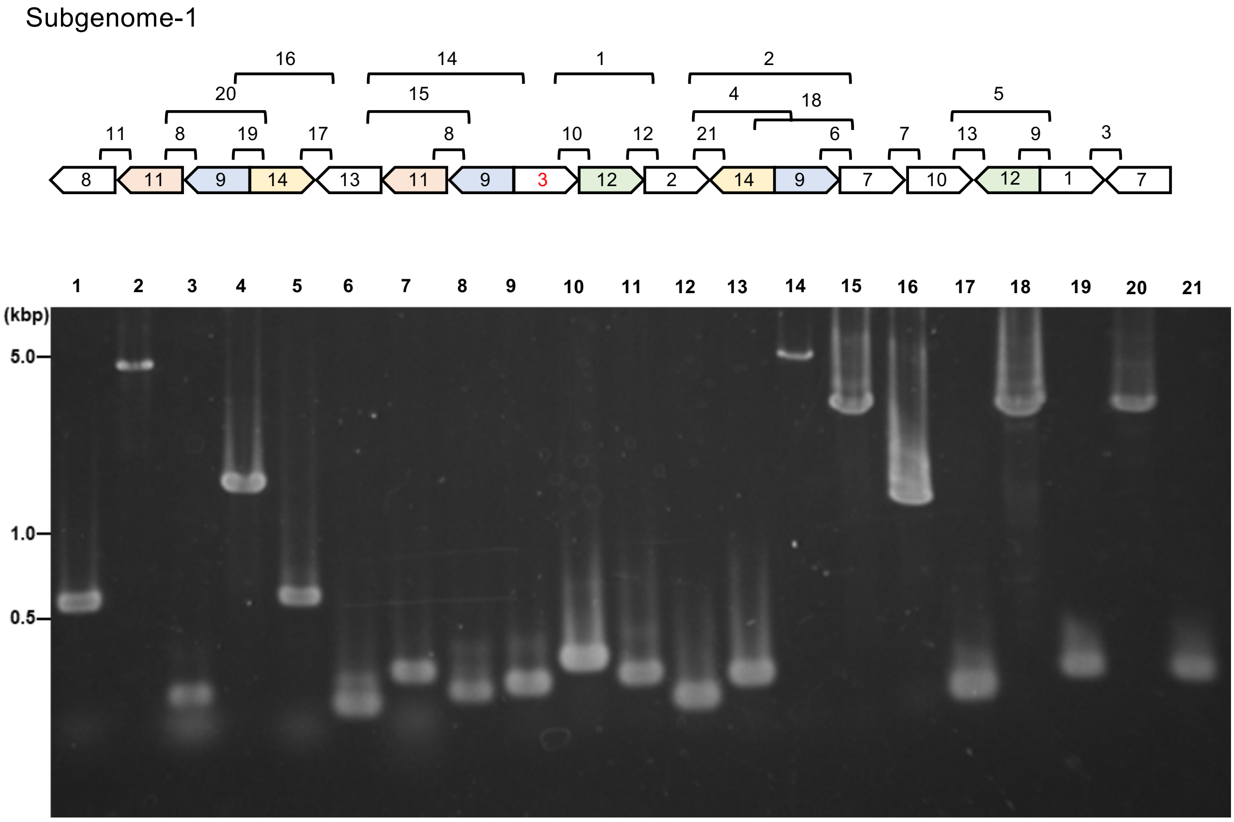

Fig. S1. Validation of the contig linkage of subgenome-1 based on PCR analysis. Lane numbers refer to the linkages between different contigs.

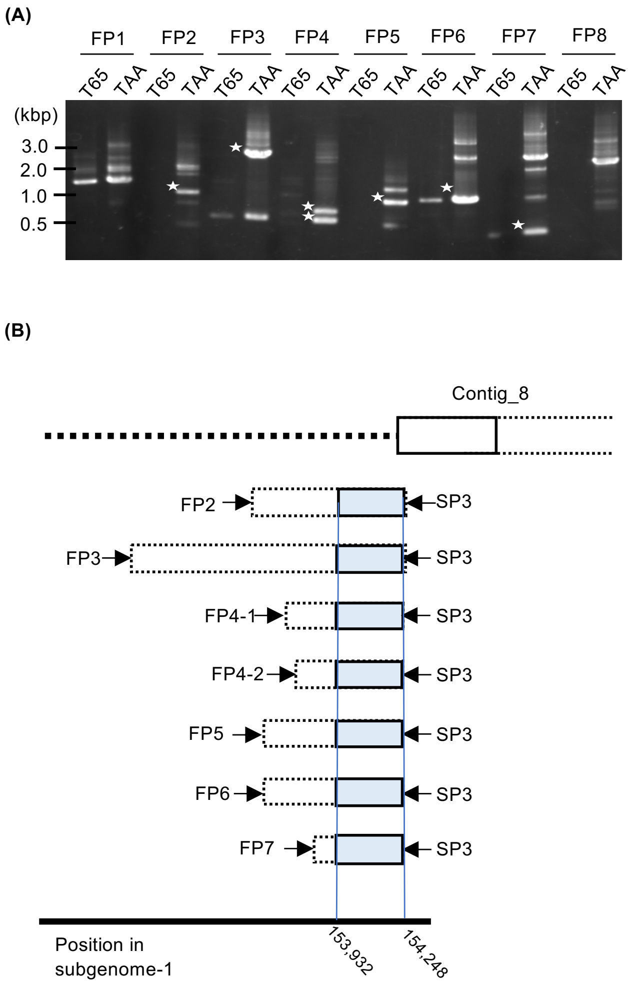

Fig. S2 Determination of the adjacent sequences of contig_8. **(A)** Amplification of the region adjacent to contig_8 using FPNI-PCR. **(B)** Schematic structures of the PCR products indicated by stars. The blue color indicates the regions in which nucleotide sequences were determined. The nucleotide sequences coincide with those in subgenome-1.

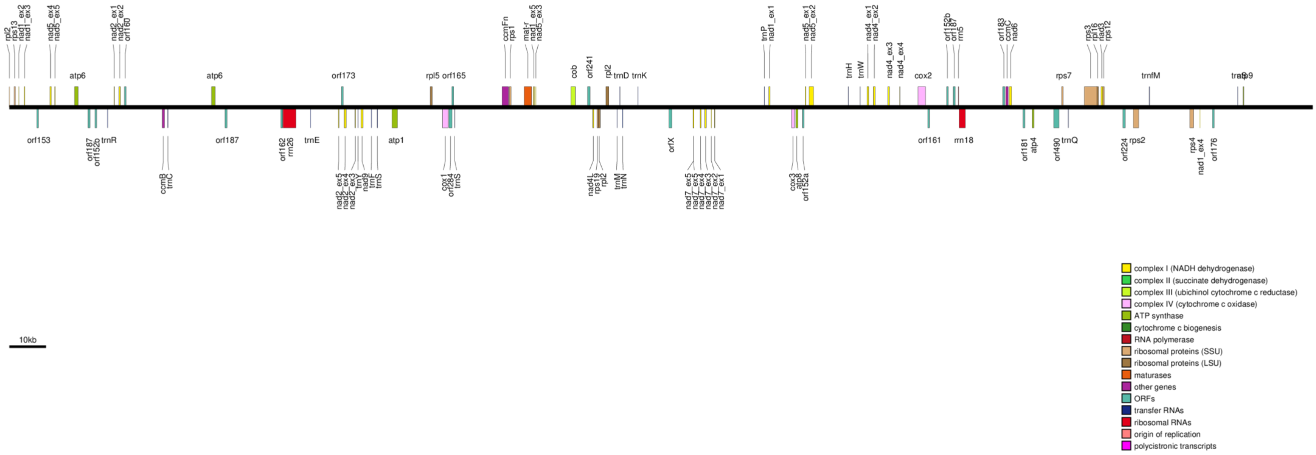

Fig. S3. A hypothetical master molecule of subgenome-1 of the TAA mitochondrial genome. The graphical representation of the genome map was generated using OGDraw software.

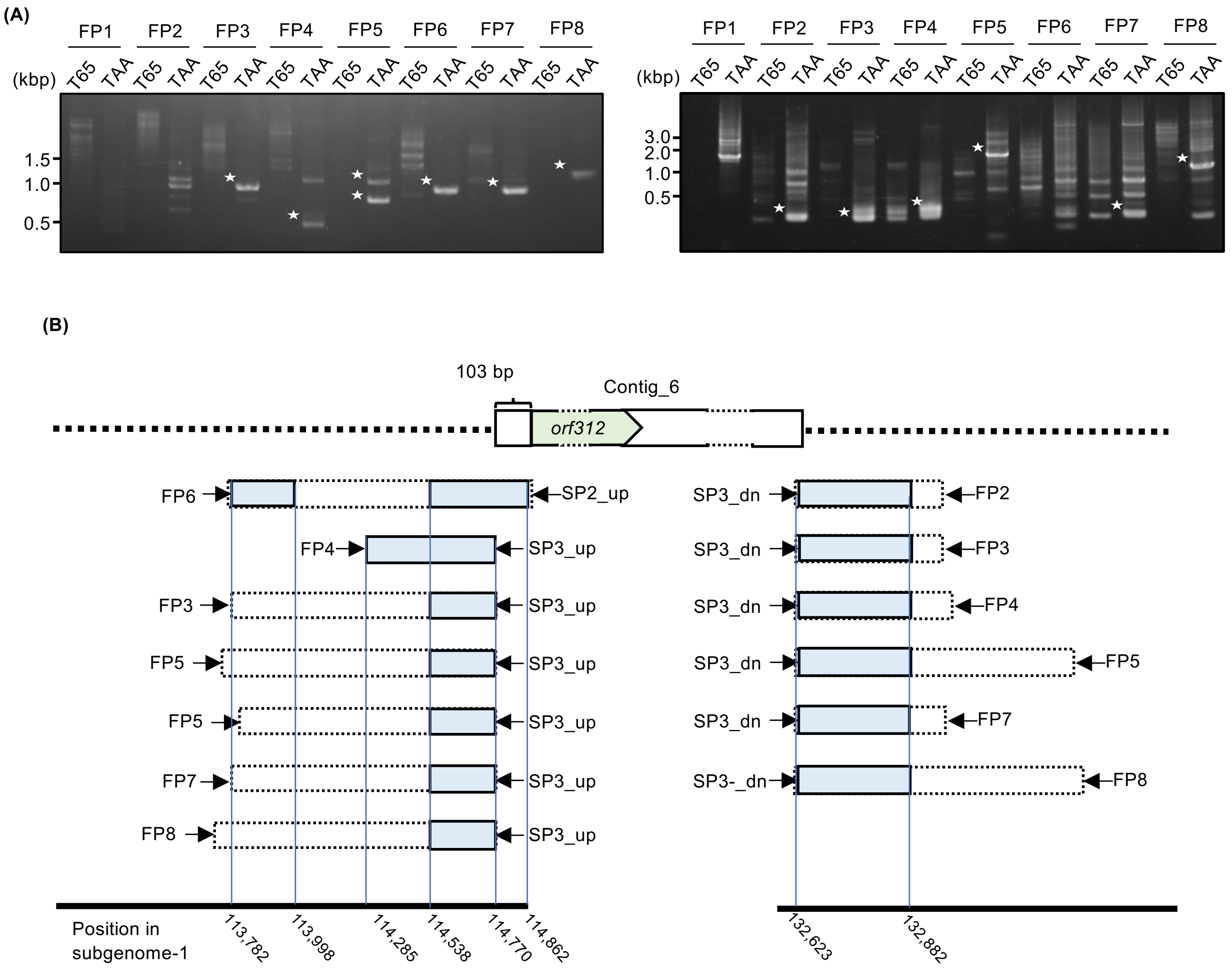

Fig. S4 Determination of the upstream and downstream regions of contig_6. **(A)** Amplification of the region adjacent to contig_6 using FPNI-PCR. **(B)** Schematic structures of the PCR products indicated by stars in panel A . The blue color indicates the regions in which nucleotide sequences were determined. The nucleotide sequences coincide with those in subgenome-1.

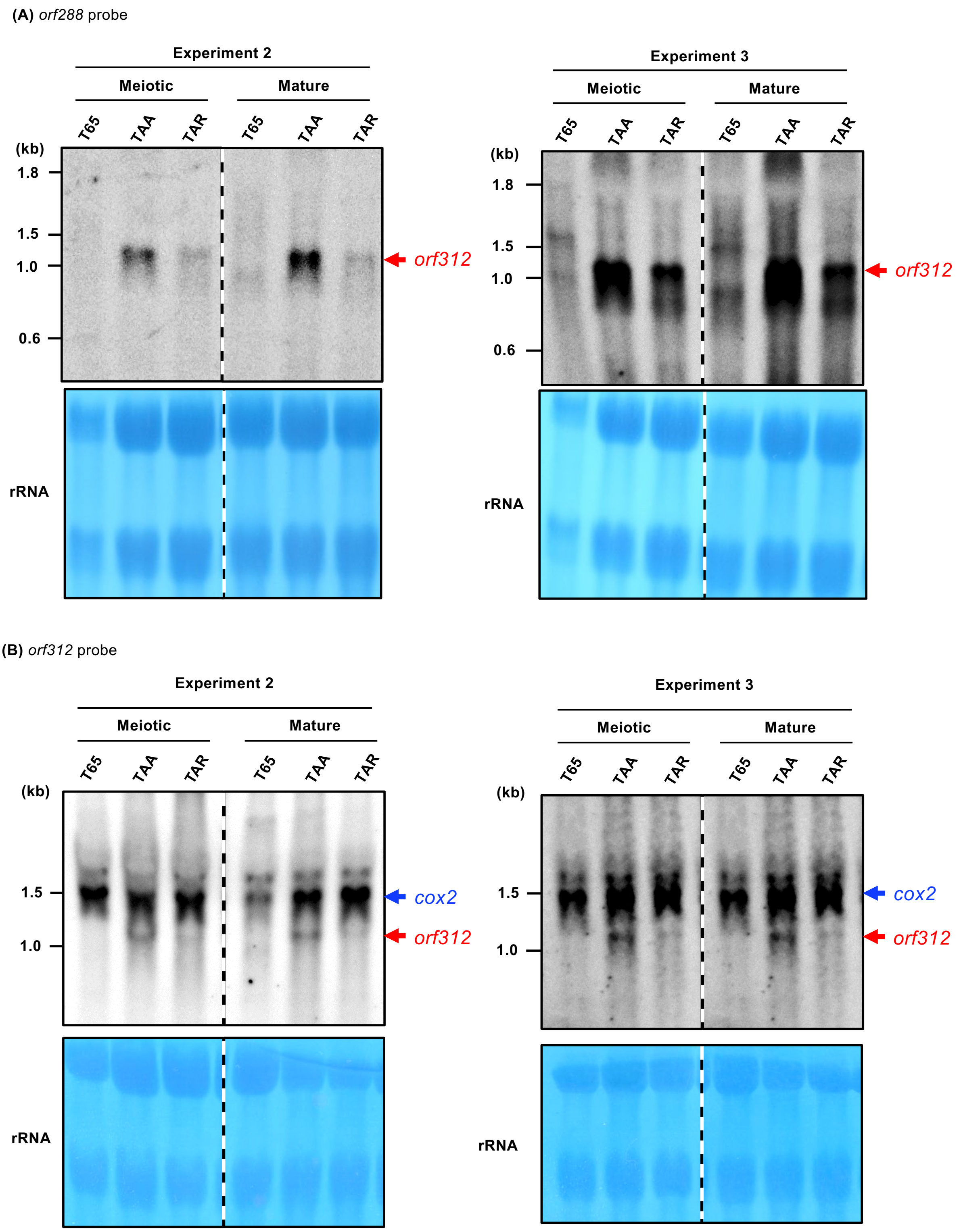

Fig. S5. Reproducibility of northern blot analysis of RNA isolated from spikelets of TAA, TAR and Taichung 65 (T65) at the meiotic stage and anthers at the mature stage. The probe is shown in Fig. 3A. Dashed lines indicate where intervening lanes were removed for clarity. Methylene blue staining of rRNA is shown as a loading control.

```
ORF288          -------MISKAERLFCRLCG--------KLVYH-----ADKLCSIMH----------DD 30
ORF312          -----MFIMIRFERACSRFIG--------KWLFS-----QDSLS--------------PD 28
WA352           MTRDRMRQSIKGRALCRAFSSRSAGGVIFKVVLKSLQERKKRISSEISSTIYFYMLCIPY 60
                          : .     : .        * :        . :.

ORF288          PIIILLILLILVFVSRMLVRALWPRSAVLTSRIFTTFVVLSSAVLSGSVAHAADNEP--- 87
ORF312          LLFLILILLILVFVSRMLVRALWPRSAVLTSRIFTTFVVLSSAVLSGSVAHAADNEP--- 85
WA352           LIFFLKISILVFLVFYRIVLPIVQMFSLLTCCSFLILILLFCLILTCLILFRLKKKEDFA 120
                 :::: * :::.:*   :* .:    ::**.  *  :::* . :*:  :  .  .::

ORF288          ------INNGAAPAAYGSGAEGPSNSALFTYTSDLAEDSVSSGRSRSTASSSFQESTGEM 141
ORF312          ------INNGAAPAAYGSGAEGPSNSALFTYTSDLAEDSVSSGRSRSTASSSFQESTGEM 139
WA352           SPPESIISIFSSIFAILMAVLASGPSVAYAMENPGPSEAGPEG-SAAPPSSSFQESTGEM 179
                      *.  ::  *   .. ... *. ::  .  ..:: ..* * :..***********

ORF288          DALMASTTPSAPRTPSGGEPSVNQPLPGEQAMPPALPVMQEAANRALPYAPYPYPVDEII 201
ORF312          DALMASTTPSAPRTPSGGEPSVNQPLPGEQAMPPALPVMQEAANRAPPYAPYPYPVDEII 199
WA352           DALMASTTPSAPRTPSGGEPSVNQPLPGEQAMPPALPVMQEAANRSPPYAPYPYPVDEII 239
                *********************************************: *************
                                                  COX11-interaction region 1

ORF288          GGDCVQSIQRRILGANLNPSAHDMQMSRIQAEDLFELKVQIIRKMATLHPSGDWMGWGAR 261
ORF312          GGDSVESIQRRLLGTNWNPSAHDIRMARMQAEDLFEVKVDIIRKMAGLHPSGDWMGWGAR 259
WA352           GGDSVQSIQRRLLGTNWNPSAHDMQMSRIQAEDLFELKVEIIRKMAGLHPSGDWMGWGAR 299
                ***.*:*****:**:* ******::*:*:*******:**:****** *************

ORF288          ALDNPRTATGEEDLAKLHKMLDDLQSRNEQSATFWRLVERVRLRADEDQNSAS 314
ORF312          ALDNPRTATGEEDLARLHQMLDDLQSGNSQSATFWLLVERVRLRAGEDQNSAS 312
WA352           ALDNPRTATGEEDLARLHQMLDDLQSRNEQSATFWRLVERVRLRADEDQNSAS 352
                ***************:**:******* *.****** *********.*******

                COX11-interaction region 2
```

Fig. S6. Comparison of the amino acid sequences of ORF288 (encoding 314 amino acids) in Nipponbare, ORF312 in TAA and WA352 in WA-CMS using ClustalW (https://clustalw.ddbj.nig.ac.jp). The COX11-interaction regions reported for WA352 are highlighted. Amino acids in ORF312 that differ from those in WA352 in these regions are indicated by red color.
